# Successful treatment of a large-sized triple-negative breast cancer: A case report

**DOI:** 10.1097/MD.0000000000050561

**Published:** 2026-09-11

**Authors:** Dong Zeng, Xiaolan Liu, Shenya Fu, Zhen Liu, Xuezhu Zhao, Luo Zhong, Xiaohui Wang

**Affiliations:** a Institute of Cancer, Sichuan Friendship Hospital, Chengdu, Sichuan, China.

**Keywords:** case report, chemotherapy, stereotactic body radiotherapy, triple-negative breast cancer

## Abstract

**Rationale:**

Triple-negative breast cancer (TNBC) is defined as the absence of estrogen receptor and progesterone receptor expression, along with a lack of human epidermal growth factor receptor 2 overexpression or amplification. Patients with TNBC exhibit a poorer prognosis, characterized by a higher propensity for metastasis to the lungs, liver, and brain, and a lower overall survival rate than other breast cancer subtypes. Primary treatment modalities for TNBC include surgery, chemotherapy, and radiotherapy. Although stereotactic body radiotherapy (SBRT) has been reported for the treatment, there is a paucity of comprehensive research in this field.

**Patient concerns:**

We report a case of successful treatment of a 38-year-old female patient with TNBC who experienced recurrence after surgery. The tumor occurred 1 year after surgery. The tumor showed rapid progression and growth.

**Diagnoses:**

Stage Ⅳ TNBC with lung metastasis

**Interventions:**

After visiting multiple hospitals, the patient finally underwent SBRT combined with chemotherapy. During SBRT implementation, an ultrahigh-dose radiotherapy modality was adopted, which is a rarely reported approach in previous studies. Subsequently, a conventional fractionated radiotherapy regimen was administered. Concurrent with radiotherapy, the patient received 2 cycles of chemotherapy.

**Outcomes:**

The patient’s tumor shrank rapidly, with minimal adverse reactions. Nine months later, the tumor remained well controlled.

**Lessons:**

Treatment of TNBC remains challenging. Given the aggressive progression of the tumor, a new radiotherapy regimen was adopted. Given that such an approach has scarcely been documented in the published literature, this case may offer novel implications for clinical practitioners.

## 1. Introduction

Globally, breast cancer is the most common type of cancer among women and the leading cause of cancer-related deaths worldwide.^[1]^ Triple-negative breast cancer (TNBC) is defined as the absence of estrogen receptor (ER) and progesterone receptor (PR) expression, along with a lack of human epidermal growth factor receptor 2 (HER2) overexpression or amplification.^[2]^ TNBC accounts for 15 to 20% of all breast cancer diagnoses.^[3]^ Patients with TNBC exhibit a poorer prognosis, characterized by a higher propensity for metastasis to the lungs, liver, and brain, and a lower overall survival rate than other breast cancer subtypes.^[4]^ Primary treatment modalities for TNBC include surgery, chemotherapy, and radiotherapy. With advancements in medical science, immunotherapy and targeted therapy are now widely utilized.^[5,6]^ Radiation therapy has been used to treat breast cancer for many years.^[7]^ Hypofractionated radiotherapy shows no significant difference in efficacy compared with conventional fractionated radiotherapy.^[8]^ Although stereotactic body radiotherapy (SBRT) has been reported for the treatment, there is a paucity of comprehensive research in this field.^[9]^ Herein, we describe the successful management of a young female patient with huge TNBC using an integrated approach of SBRT and chemotherapy, which yielded an excellent clinical response. Our experience with this case may provide useful perspectives for colleagues engaged in managing similar malignancies.

## 2. Case report

The patient was a 37-year-old married woman from a rural area. She had no family history of malignancy, hypertension, diabetes mellitus, infectious diseases, or other comorbidities. In April 2024, the patient accidentally palpated a pea-sized nodule on the lateral aspect of her left breast. A surgical excision biopsy was performed at the First Affiliated Hospital of Sun Yat-sen University in Guangzhou, and the pathology report dated April 9, 2024, confirmed invasive breast carcinoma. Immunohistochemistry results obtained on April 24, 2024, showed ER (−), PR (−), human epidermal growth factor receptor‑2 (0), and marker of proliferation Ki‑67 positivity in approximately 50% of the tumor cells. The patient underwent the following surgical procedures: left nipple-sparing subcutaneous mastectomy, left axillary sentinel lymph node biopsy, unilateral breast prosthesis implantation, right breast mastopexy, and fasciocutaneous flap revision. Postoperatively, she received 1 cycle of chemotherapy with epirubicin 140 mg combined with cyclophosphamide 0.94 g. However, owing to intolerable side effects, the patient did not continue chemotherapy or radiotherapy. Only 14 months later, the patient experienced tumor recurrence. In June 2025, the patient noticed a new mass on the left chest wall measuring approximately 21 × 10 mm. A chest wall biopsy revealed tumor cells, confirming postoperative recurrence of breast cancer. Due to financial hardship, the patient did not seek timely medical attention. The mass grew rapidly in August 2025. Given the large size of the tumor, tissue specimens were readily obtainable. A repeat core needle biopsy was performed at Southwest Hospital in Chongqing in September 2025, confirming the diagnosis of TNBC. Immunohistochemistry results were as follows: cytokeratin 5/6 (+), E-cadherin (+), P63 (−), cytokeratin 14 (+), synaptophysin (−), ER (−), PR (−), marker of proliferation Ki‑67 (80%+), cluster of differentiation 8 (−), forkhead‑box protein C1 (strongly positive in 90% of cells), and HER2 (0). Differential diagnosis should be performed to distinguish it from HER2-positive or hormone receptor-positive breast cancer.

**Figure 1.**
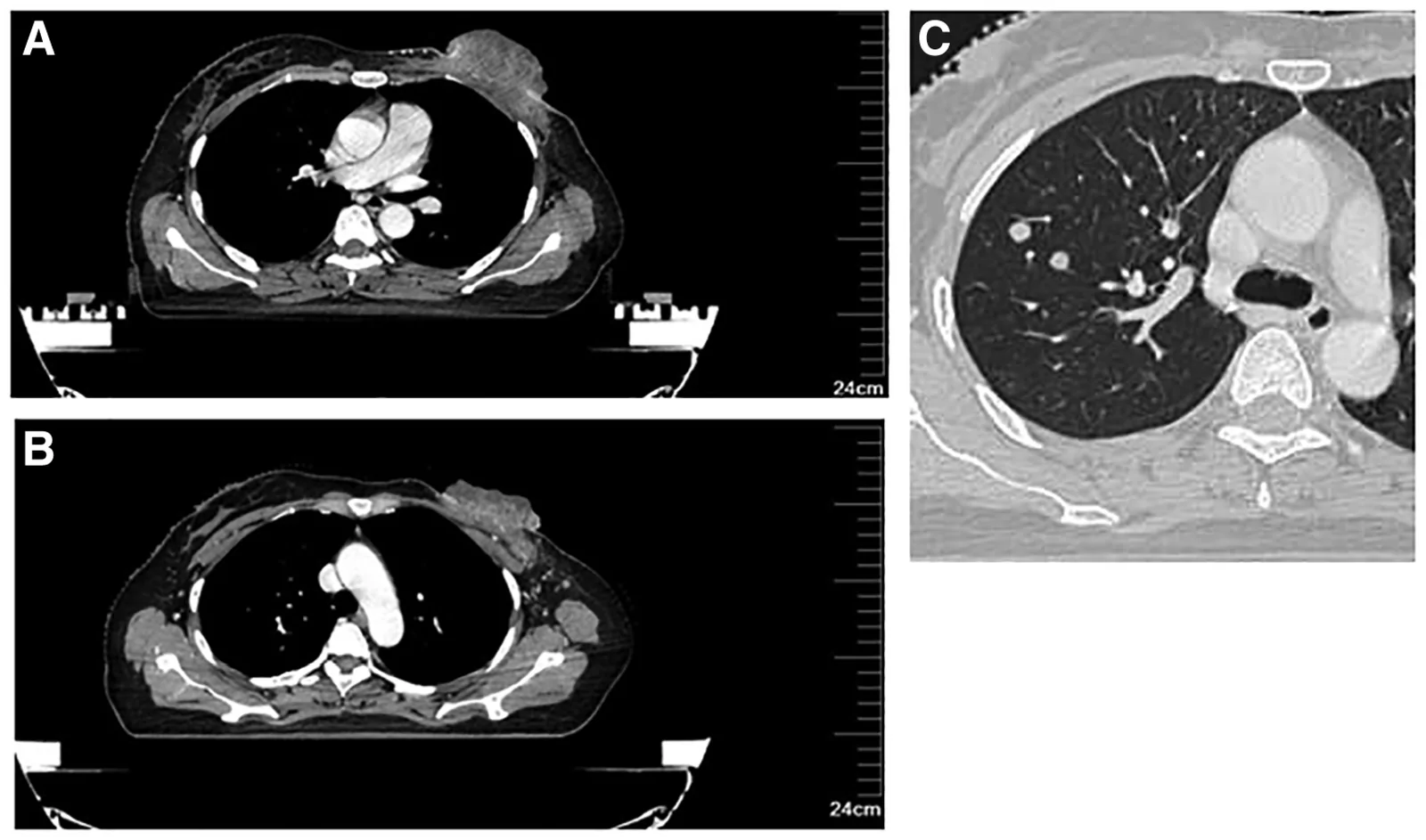
Contrast-enhanced CT revealed (A) a large tumor in the left breast; (B) a large neoplasm in the left breast at the aortic arch level; (C) metastatic lesions in the upper lobe of the right lung. CT = computed tomography.

**Figure 2.**
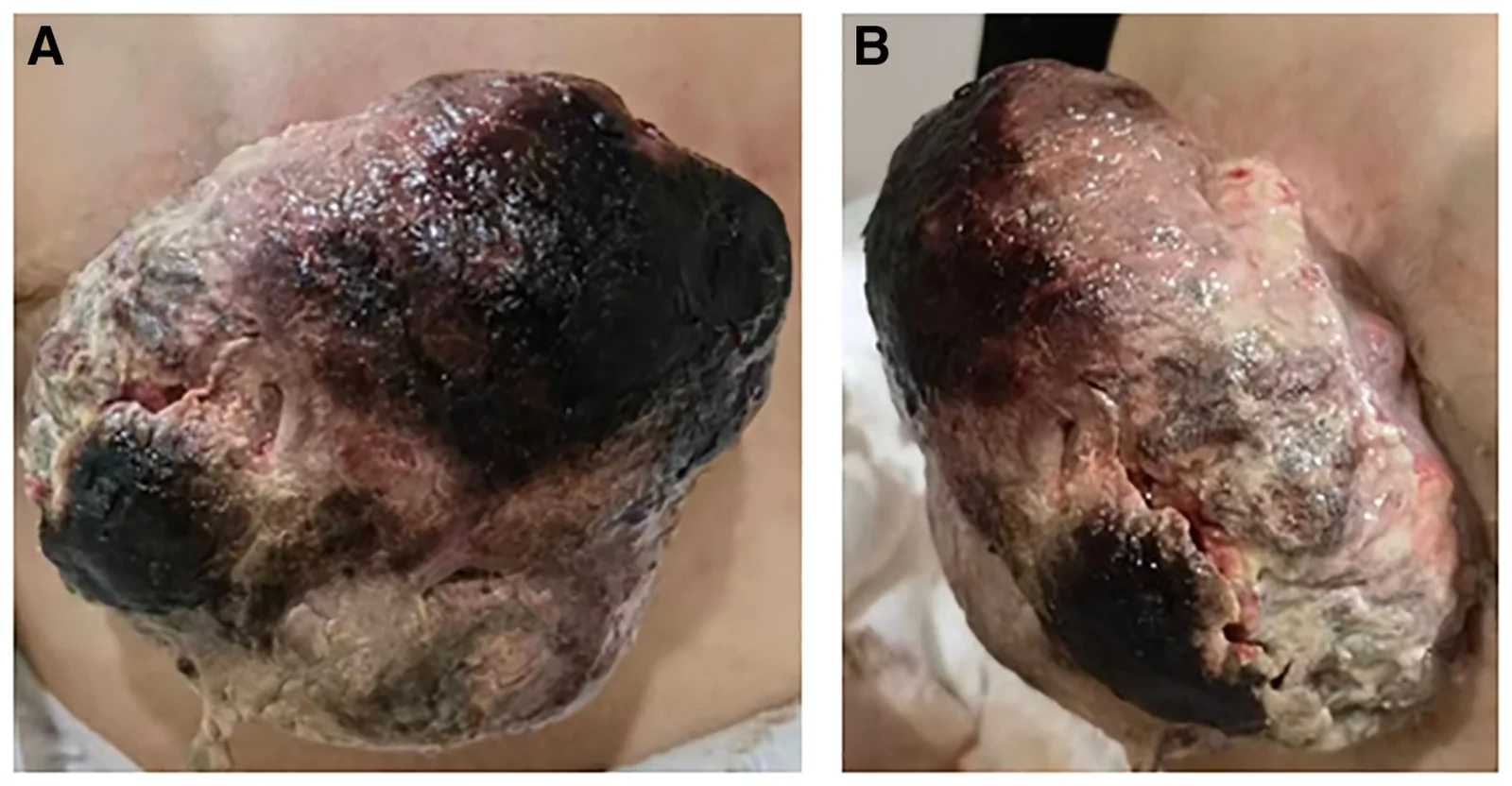
Tumors on (A) anteroposterior view and (B) lateral view.

Subsequently, the patient was admitted to West China Hospital of Sichuan University. A breast ultrasound on October 17, 2025, showed a large solid mass in the left chest wall, suggestive of carcinoma; a solid nodule in the right breast, with an intraductal lesion to be excluded (breast imaging reporting and data system category 4a); and enlarged left axillary lymph nodes with abnormal morphology in some cases. A contrast-enhanced thin-section chest computed tomography scan on October 21, 2025, demonstrated a large soft tissue mass in the left chest wall and increased and partially enlarged left axillary lymph nodes, suggesting recurrent left breast cancer with chest wall metastasis and possible left axillary lymph node metastasis (Figs. 1A and 1B). Imaging examination revealed multiple metastases in the bilateral lungs (Fig. 1C). Gross examination revealed a large mass in the left breast accompanied by localized necrosis in the lesion (Figs. 2A and 2B). The neoplasm had 3-dimensional dimensions of approximately 11 cm × 9 cm × 8 cm. Severe anemia developed due to massive hemorrhage from the left breast mass, with a lowest hemoglobin level of 63 g/L. The patient was diagnosed with stage IV tumor. Advanced TNBC typically confers a poor prognosis.

**Figure 3.**
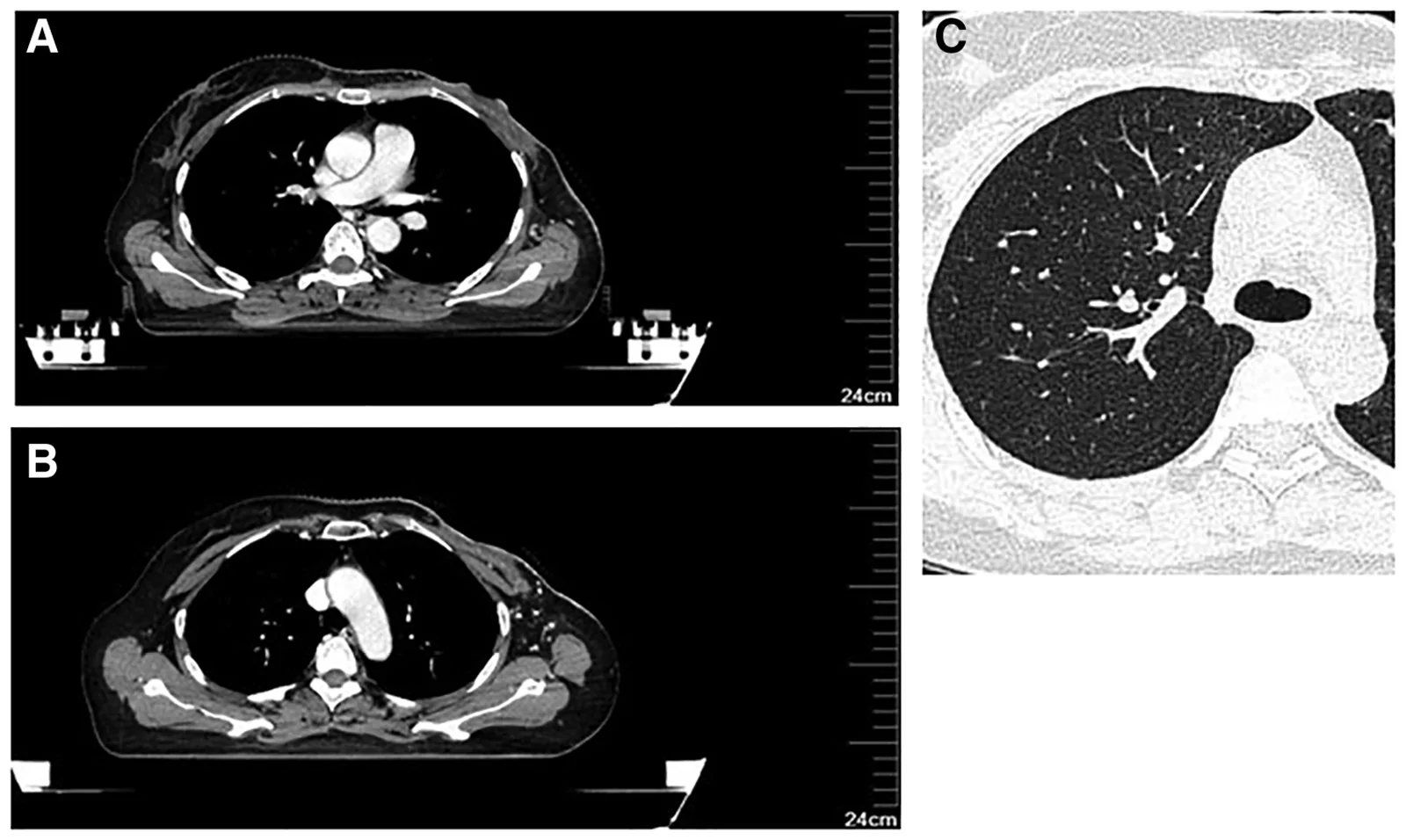
(A) Contrast-enhanced CT revealed marked regression of the (A) tumor; (B) tumor at the aortic arch level; (C) tumor at the lung. CT = computed tomography.

**Figure 4.**
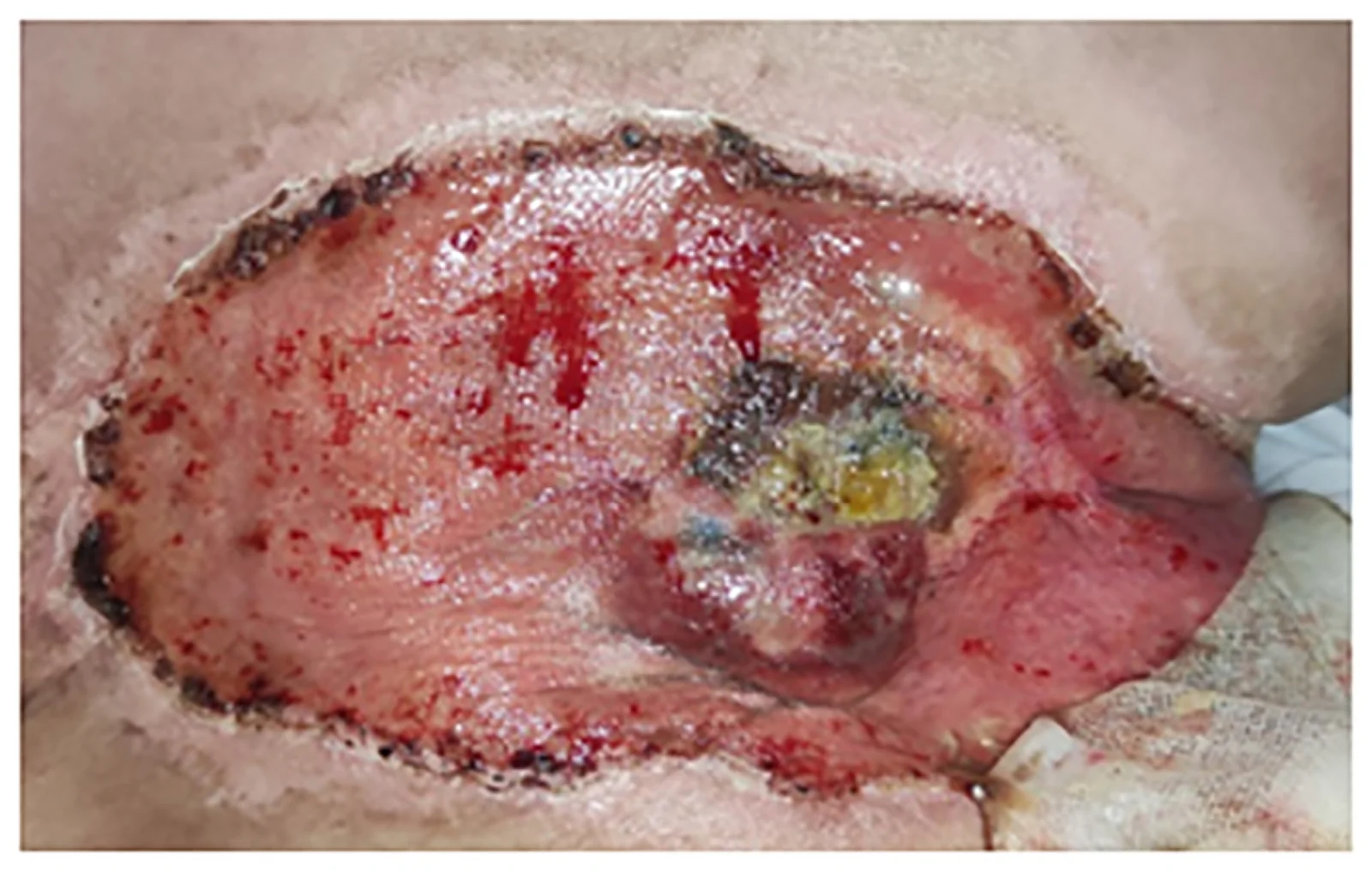
The tumor showed a significant reduction in size.

Thereafter, SBRT was administered to the left chest wall mass at the following dose: 1200 cGy to the planning gross tumor volume in the first fraction, followed by 4 fractions of 800 cGy each. Radiotherapy was also delivered to the chest wall region at a dose of 41 Gy in 23 fractions (41 Gy/23 f) to the planning clinical target volume (PCTV) and to the axillary lymph node region at a dose of 49.2 Gy in 23 fractions (49.2 Gy/23 f) of planning gross tumor volume-N. intensity-modulated radiation therapy technology was administered. Concurrent with radiotherapy, the patient received 2 cycles of chemotherapy, which included docetaxel at a dose of 115 mg, epirubicin at 70 mg, and cyclophosphamide at 0.75 g per cycle. Owing to the massive tumor burden, the patient faced risks including hemorrhage, infection, and myelosuppression throughout treatment. Following these treatments, a chest computed tomography revealed significant shrinkage of the mass, indicating a favorable treatment response (Fig. 3A and 3B). The tumors in the lungs also shrunk (Fig. 3C). The patient’s mass showed rapid regression (Fig. 4). Few adverse reactions were noted in the patient. The patient tolerated the treatment well. No notable adverse events were observed, and the patient’s quality of life improved progressively. No unanticipated events were noted. As of July 2026, the patient remains alive with a well-controlled tumor.

## 3. Discussion

As a highly aggressive subtype of breast cancer, TNBC is distinguished by its pronounced drug resistance, rapid clinical progression, poor clinical outcomes, and absence of clearly defined molecular therapeutic targets.^[10]^ Compared with luminal A/B and HER2+ tumors, TNBC has poorer overall survival.^[11]^ Based on molecular profiling, the subtypes include 2 basal-like (BL) subtypes (BL1 and BL2), 1 immunomodulatory subtype, 1 mesenchymal subtype, 1 mesenchymal stem cell-like subtype, and 1 luminal androgen receptor subtype.^[12]^ A wide array of therapeutic modalities are available for the management of TNBC, including surgery, chemotherapy, radiotherapy, immunotherapy, and molecular-targeted therapy. Surgery plays a crucial role in the development of early-stage TNBC. BCT and mastectomy are viable treatment options.^[13]^ Currently, chemotherapy is the only systemic treatment modality for TNBC; however, an optimal therapeutic regimen has not yet been established. Taxane- and anthracycline-based regimens represent the mainstay of treatment for TNBC, and platinum-based chemotherapy has demonstrated favorable efficacy in neoadjuvant and metastatic settings.^[14]^ For TNBC, neoadjuvant chemotherapy yields a higher pathological complete response (pCR) rate than hormone receptor-positive, HER2-negative breast cancer, with rates ranging from 28 to 30% versus 6.7%. pCR rates vary by TNBC subtype; the BL1 subtype has the highest pCR rate at 52%, while the BL2, mesenchymal, and luminal androgen receptor-positive subtypes have the lowest rates.^[15,16]^ Current clinical guidelines recommend neoadjuvant systemic therapy as the first-line treatment for patients diagnosed with stage II–III TNBC.^[17]^ Several studies have explored the potential benefits of adjuvant therapy following neoadjuvant chemotherapy. For patients who have not received neoadjuvant chemotherapy (with the possible exception of those with rare histological subtypes), the European Society for Medical Oncology guidelines recommend adjuvant chemotherapy.^[18]^ The selection of an initial systemic chemotherapy regimen should be personalized based on multiple factors, including the tumor burden, disease progression rate, patient performance status, prior chemotherapy history, and individual patient preferences.^[19]^ Anthracycline-taxane chemotherapy regimens are recommended as the initial treatment options. We adhered to this principle in this case.

Radiotherapy as a single modality significantly improves overall survival and disease-free survival, regardless of the tumor molecular subtype. This highlights the importance of radiotherapy in all clinical contexts.^[20]^ Radiotherapy has been an established and widely used treatment modality for breast cancer for several decades. Preoperative radiotherapy is an effective strategy for enhancing the pCR rate in patients with locally advanced breast cancer.^[21]^ For patients with TNBC, radiotherapy reduces the risk of locoregional recurrence and improves overall survival compared with non-radiotherapy patients. Adjuvant radiotherapy to the breast is typically administered at a dose of 50 Gy in 25 fractions or 42.5 Gray in 16 fractions.^[22,23]^ In cases where surgical resection is not feasible, radiotherapy is a critical therapeutic alternative.^[24]^ In recent years, there has been increasing interest in the application of high-dose focused radiotherapy, including SBRT, as a therapeutic approach for metastatic lesions.^[25]^ SBRT is widely used for oligometastatic breast cancer and is a well-tolerated treatment with limited toxicity. As reported in a systematic review, SBRT doses range from 24 to 60 Gray in 1 to 10 fractions, based on lesion location and size.^[26]^ In this study, we increased the single-fraction SBRT dose to 12 Gy, achieving rapid tumor shrinkage. Notably, such an ultrahigh-dose therapeutic approach has not been documented in any previously published studies. However, a subset of patients with TNBC exhibits resistance to RT, leading to recurrence and poor treatment outcomes. The combination of radiotherapy and anti-programmed cell death protein 1/programmed death‑ligand 1 (PD-L1) inhibitors has shown significant therapeutic efficacy in clinical oncology research.^[27]^ Radiotherapy plays a crucial role in modulating the immune microenvironment of TNBC by altering the differentiation and function of T cells, potentially enhancing the immune response.^[28]^ In addition to traditional radiotherapy, ongoing efforts to optimize radiotherapy techniques, such as the introduction of proton therapy, can improve therapeutic efficacy while reducing side effects.^[29]^

Currently, precision and individualized therapy are key focuses of cancer treatment, and tumor-targeted therapy has revolutionized cancer treatment modalities. Poly adenosine diphosphate-ribose polymerase inhibitors are successful examples of precision medicine. Olaparib, rucaparib, niraparib, and talazoparib have been approved for the treatment of BRCA-mutated breast cancer.^[30]^ BRCA1/2 gene mutations are detected in nearly 20% of TNBC patients, with the highest prevalence reported in Black and Hispanic populations.^[31]^ A subgroup of androgen receptor-positive TNBC accounts for approximately 30% of all TNBC cases. Data have shown that antiandrogen drugs can confer certain benefits to patients.^[32]^ Trop-2-targeted antibody-drug conjugates, formed by conjugating Trop-2 antibodies with cytotoxic drugs, have shown encouraging therapeutic effects in metastatic TNBC.^[33]^ A phase 3 clinical trial demonstrated that in previously treated TNBC patients, sacituzumab tirumotecan yielded a median progression-free survival of 6.7 months, in contrast to merely 2.5 months with chemotherapy.^[34]^

Immunotherapy has been extensively used for the clinical management of TNBC.^[35]^ TNBC is characterized by high PD-L1 expression levels and high tumor mutational burden, making it more suitable for immunotherapy than conventional therapies.^[36]^ KEYNOTE-522 findings indicate that the combination of chemotherapy and pembrolizumab yields a significant pCR advantage in patients with stage II and III TNBC.^[37]^ In patients with early-stage TNBC, the administration of neoadjuvant therapy consisting of atezolizumab in combination with nedaplatin and anthracycline-based chemotherapy regimens resulted in a marked increase in the pCR rate, with a favorable and acceptable safety profile.^[38]^ With a PD-L1 combined positive score of 5, this patient is expected to derive greater clinical benefit from subsequent immunotherapy. According to a multicenter phase II trial, the PD-L1/cytotoxic T‑lymphocyte‑associated antigen 4 bispecific antibody KN046 in combination with nab-paclitaxel as first-line therapy for patients with metastatic TNBC yielded an objective response rate of 44.0% (95% confidence interval: 24.4–65.1%) and a median progression-free survival of 7.33 months (95% confidence interval: 3.68–11.07 months).^[39]^ Preclinical and clinical advances have been made in chimeric antigen receptor-T cell therapy for TNBC; however, more data are needed to demonstrate its antitumor efficacy.^[40]^ Immunotherapy will also be incorporated into the subsequent treatment process.

However, the patient’s overall survival remains shorter than 1 years, and further treatment and follow-up surveillance are warranted. More cases are needed for further verification.

## 4. Conclusion

We presented the initial treatment regimen for a young female patient diagnosed with bulky TNBC. After radiotherapy combined with chemotherapy, excellent antitumor effects were achieved. Notably, an ultrahigh-dose radiotherapy strategy was employed in this case. In July 2026, the disease remained well controlled. The patient was grateful for the treatment received. The patient hereby grants authorization for the release of all medical records, images, and related materials. Given that such an approach has scarcely been documented in the published literature, this case may offer novel implications for clinical practitioners.

## Acknowledgments

We would like to express our gratitude to the patient for granting permission to use their clinical data in this paper and for the publication of this research.

## Author contributions

**Data curation:** Dong Zeng.

**Formal analysis:** Dong Zeng, Xiaolan Liu.

**Funding acquisition:** Zhen Liu.

**Methodology:** Dong Zeng, Shenya Fu.

**Project administration:** Luo Zhong, Xiaohui Wang.

**Supervision:** Xuezhu Zhao.

**Writing – original draft:** Dong Zeng.

**Writing – review & editing:** Dong Zeng, Xiaolan Liu, Xiaohui Wang.
